# Probing spatial heterogeneity in silicon thin films by Raman spectroscopy

**DOI:** 10.1038/s41598-017-16724-4

**Published:** 2017-11-29

**Authors:** Hideyuki Yamazaki, Mitsuo Koike, Masumi Saitoh, Mitsuhiro Tomita, Ryo Yokogawa, Naomi Sawamoto, Motohiro Tomita, Daisuke Kosemura, Atsushi Ogura

**Affiliations:** 1 Corporate Research & Development Center, Toshiba Corporation, 1 Komukai Toshiba-cho, Saiwai-ku, Kawasaki, 212-8582 Japan; 2 School of Science and Technology, Meiji University, 1-1-1 Higashimita, Tama-ku, Kawasaki, 214-8571 Japan

## Abstract

Raman spectroscopy is a powerful technique for revealing spatial heterogeneity in solid-state structures but heretofore has not been able to measure spectra from multiple positions on a sample within a short time. Here, we report a novel Raman spectroscopy approach to study the spatial heterogeneity in thermally annealed amorphous silicon (a-Si) thin films. Raman spectroscopy employs both a galvano-mirror and a two-dimensional charge-coupled device detector system, which can measure spectra at 200 nm intervals at every position along a sample in a short time. We analyzed thermally annealed a-Si thin films with different film thicknesses. The experimental results suggest a correlation between the distribution of the average nanocrystal size over different spatial regions and the thickness of the thermally annealed a-Si thin film. The ability to evaluate the average size of the Si nanocrystals through rapid data acquisition is expected to lead to research into new applications of nanocrystals.

## Introduction

Silicon nanocrystal (SiNC) films have attracted much attention for applications such as optoelectronic, photonic, and photovoltaic devices^1^. In particular, the fact that the optical and electronic transport properties of SiNC semiconductors are strongly related to the spatial heterogeneity of the films is of considerable interest both scientifically and technologically^2–5^. On the other hand, the growth of SiNCs by thermal annealing leads to changes in the spatial heterogeneity. Therefore, high-performance devices require precise control of the average SiNC size distribution, which has made characterization of the average SiNC size increasingly important. Practical techniques for probing the average SiNC size distribution need to be non-destructive, time efficient, and convenient. Raman spectroscopy has been shown to be a powerful tool that meets all of these requirements^6^. However, measurement at all positions on a sample by conventional Raman instruments requires long acquisition times^7^ and is not suitable for statistical analysis.

**Figure 1:**
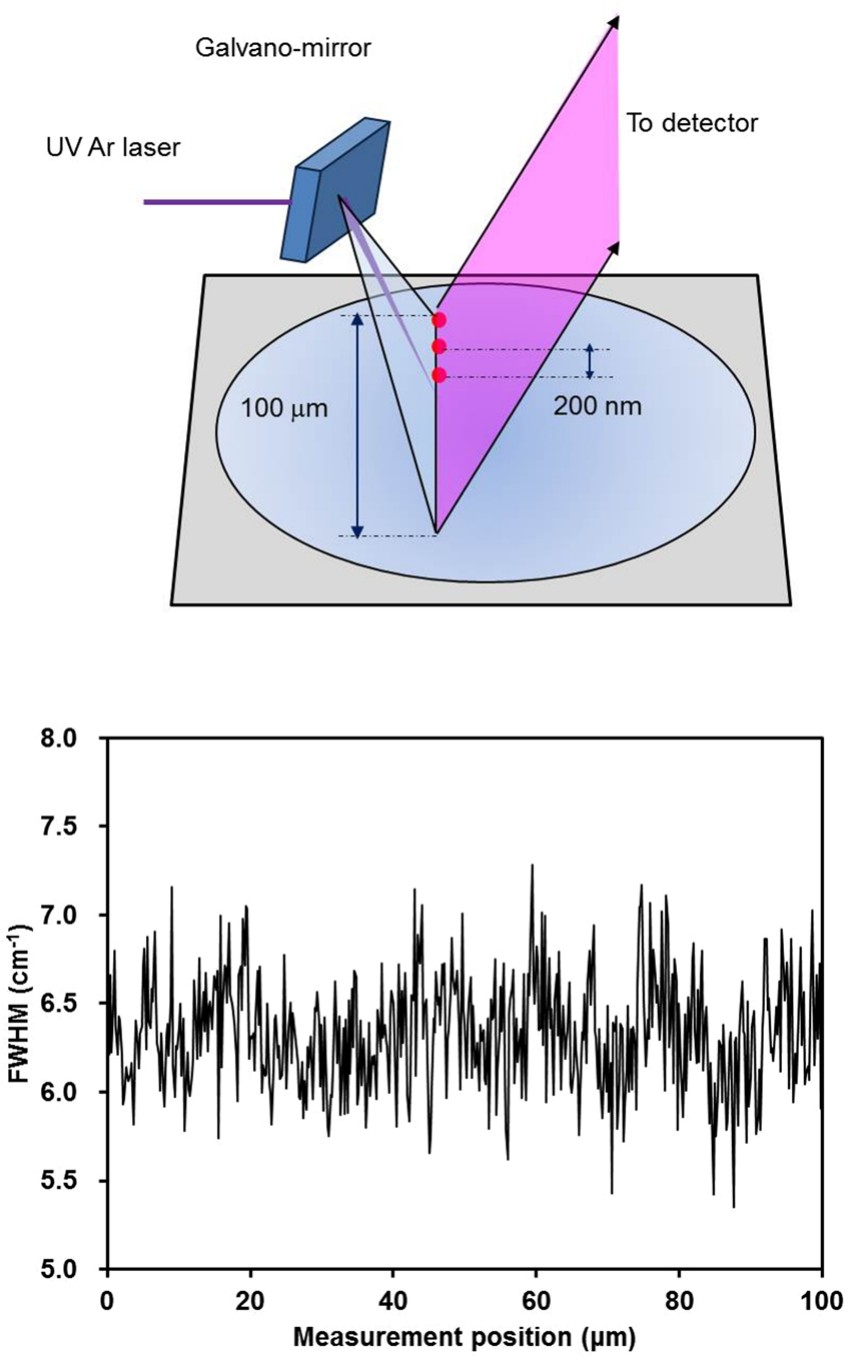
(**a**) Schematic showing multipoint Raman measurement and (**b**) example of FWHM values of the Raman spectra obtained from a 100-nm-thick film as a function of measurement position. The number of measurement points was 512. The lines joining the data points are a guide for the eyes.

To overcome these problems, the combination of a quasi-line excitation source obtained with a galvano-mirror and a two-dimensional charge-coupled device (CCD) detector system is used in our state-of-the-art Raman spectrometer^8,9^. This quasi-line light source has a uniform intensity along the line, enabling high-speed measurement. Moreover, we can obtain both spatial and energy information simultaneously using a two-dimensional CCD pixel array. Thus, we can obtain spectra at 200 nm intervals at every position along a sample. This technique is therefore expected to allow for statistical investigation of the average SiNC size by rapid measurements (Fig. 1). Another advantage of this Raman spectroscopy system is that the quasi-line-shaped excitation-light source offers higher spatial resolution^9^ than can be obtained from conventional Raman spectroscopy, in which the spatial resolution is limited by the spot size of the light source. In this work, we investigate the influence of film thickness on the average SiNC size distribution over different spatial regions in thermally annealed amorphous silicon (a-Si) thin films to demonstrate the application of our Raman spectroscopy technique. First, we confirm the quantitative accuracy of the average SiNC size obtained by Raman spectroscopy compared with both X-ray diffraction (XRD) and plan-view dark-field transmission electron microcopy (TEM). These experiments were performed using the following procedures. Raman and XRD measurements were first carried out on the same sample (100-nm-thick thin film), and then, the plan-view TEM observation was performed. The sample thickness of 100 nm was chosen by considering the penetration depth of X-rays. Next, the influence of film thickness on the average SiNC size distribution over different spatial regions in thermally annealed a-Si thin films was investigated.

## Results and Dicussion

**Figure 2:**
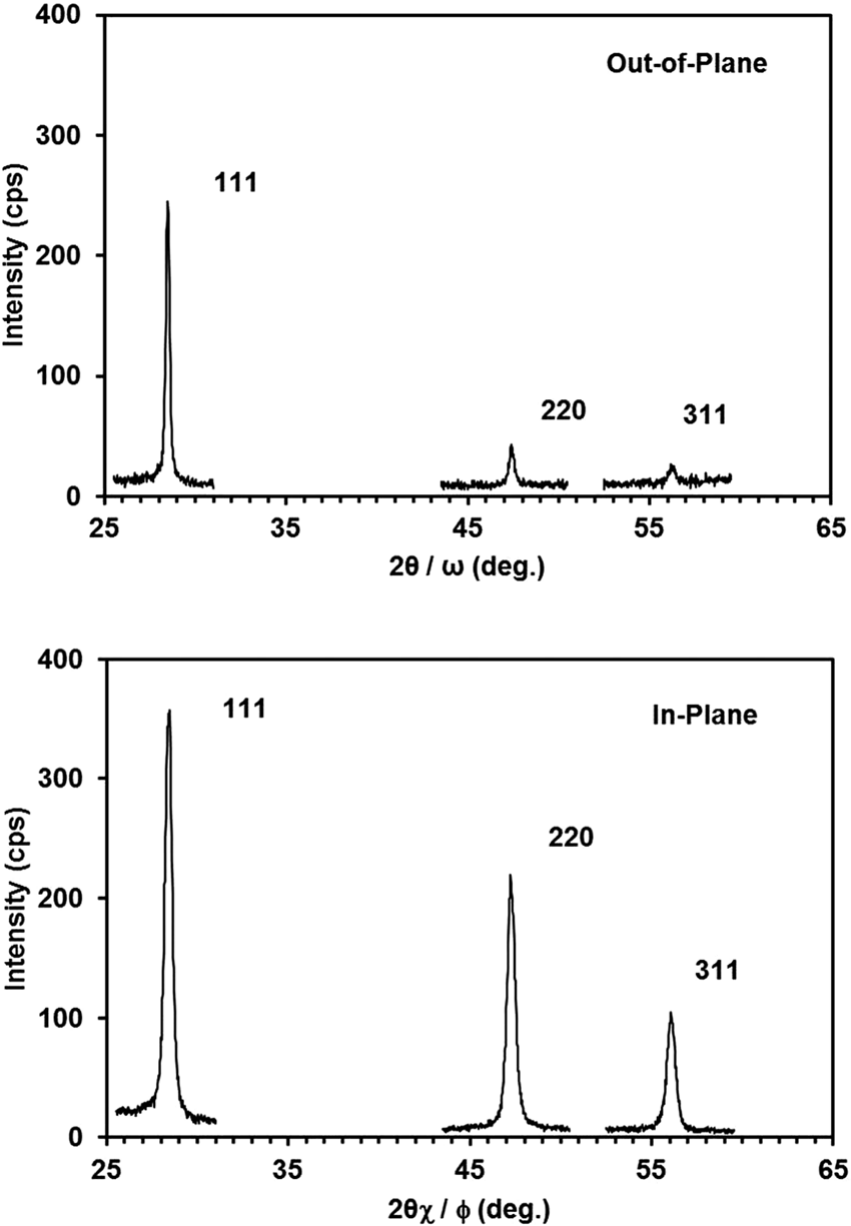
(**a**) Out-of-plane and (**b**) in-plane XRD patterns of a 100-nm-thick a-Si thin film thermally annealed at 700 °C for 10 min. The vertical dotted line at 2*θ* = 28.4° is the position of the Si (111) peak in the thin films, which are assumed to be unstrained^26^.

**Figure 3:**
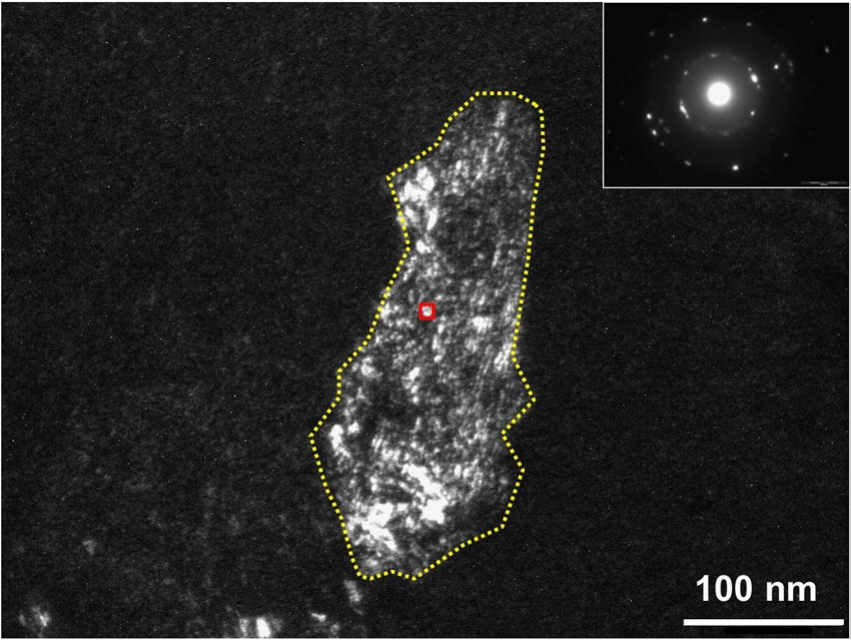
Dark-field plan-view TEM micrograph of a 100-nm-thick a-Si thin film thermally annealed at 700 °C for 10 min. The related diffraction pattern is shown in the inset. The Si grain and one of the SiNCs are demarcated with a yellow dotted line and a red solid line, respectively, to guide the eyes. A Si grain is made up of many SiNCs.

From the Raman spectroscopy analysis of the film sample annealed at 700 °C for 10 min (see Fig. 1(b)), we can obtain the size of SiNCs with a given diameter by using the phonon confinement model of Ke, Feng, and Huang (hereinafter, the KFH model)^10^. We deduced an average diameter of 11.7 ± 0.7 nm (average ± standard deviation). In-plane and out-of-plane grazing-incident XRD measurements were performed to obtain information about the crystal-structure parameters, including size. The combination of in-plane and out-of-plane techniques is useful for studying film structures in two directions, namely, perpendicular and parallel to the surface^11^. Figure 2 shows the out-of-plane and in-plane XRD patterns obtained from the same sample. The XRD spectrum from the out-of-plane configuration suggests that the [111] direction is the preferential growth direction of the film, whereas the in-plane scans show no preferred orientation. The crystallite diameter of the SiNCs was calculated from the (111) peak (Table 1). It can be seen that the SiNC size differs in the out-of-plane and in-plane XRD analyses. These results are presumably due to the growth of columnar-like crystals. It is well known that the annealing of a-Si films leads to crystallization through the nucleation and growth of crystals^12^. Furthermore, films grown on a SiO_2_ layer exhibit a polycrystalline structure with no apparent preferred orientation of the crystallites. Thus, in this experimental sample, in which SiO_2_ is present between the thermally annealed a-Si film and the crystalline Si substrate, the crystal orientation at the early stage of thermal annealing is determined by the orientation of the initial nuclei. Films deposited by using the low-pressure chemical vapor deposition process are nearly amorphous and include point defects, which act as nucleation sites^13,14^. As mentioned above, the data presented in Fig. 2 suggest that the crystals grow predominantly along the [111] direction. Once the {111} facet forms, the silicon nanocrystals will preferentially grow along the <111> direction into columnar-like crystals until they reach the surface and then continue to grow laterally with multiple orientations. It can be concluded that the average SiNC size is very large in the out-of-plane direction because the experimental sample is undergoing crystal growth. Figure 3 shows the dark-field plan-view TEM images of the same sample. The film is partially crystallized, as seen from the electron diffraction pattern shown in the inset. Bright spots in the grains show SiNCs with roughly spherical and elliptical shapes. The SiNC size (*d*) was calculated from equation (1):

1 $$d=\sqrt{\pi ab}/2$$

where *a* and *b* are the lengths of the long and short axes, respectively. Table 1 lists the average sizes obtained by Raman spectroscopy, XRD analysis and TEM observations. For XRD analysis, we focus on the results obtained from the in-plane XRD scan mode for comparison with the Raman results, because the out-of-plane XRD results, shown in Table 1, cannot be obtained by the Raman spectroscopy technique owing to the information depth of approximately 5 nm in this experiment. Although the information depth is shallow, the Raman technique offers benefits in the structural characterization of thin films. For example, the Raman technique is useful for application to the structural analysis of polysilicon thin-film channels (e.g., 5 to 10 nm thick) for the development of high-performance memory devices^15^. One should keep in mind that small discrepancies in the obtained values are primarily attributable to the different physical principles of the three techniques. One difference is the information depth of each technique. The out-of-plane and in-plane XRD measurements in these experiments can obtain information at depths of up to 80 nm, whereas the depth of TEM equals the sample thickness of approximately 100 nm. In contrast, the Raman technique probes very close to the surface to a depth of 5 nm, as discussed in ref.^9^. Another difference is the size measurement error in TEM observations. Small SiNCs may be overlooked. If small SiNCs are covered by large SiNCs in the transmission direction, the determination of small SiNC sizes can be challenging. However, another factor is the difference in the SiNC shape model among the three techniques. The SiNCs are assumed to be spherical in the Raman and XRD analyses, whereas an elliptical approximation was employed in the TEM observations. Even considering the above issues, the SiNC sizes obtained from the three techniques were in satisfactory agreement. Note that the Raman technique is unable to obtain information about the relationship between SiNC size and spacing. However, from the plan-view dark-field TEM images, it can be postulated that each spectrum contains information for approximately 50 to 70 SiNCs depending on the nature of the sample.

**Table 1:** Average SiNC sizes determined from Raman spectroscopy, XRD ((111) diffraction line), and plan-view TEM on a 100-nm-thick a-Si film thermally annealed at 700 °C for 10 min. The average size obtained from Raman spectroscopy and XRD shows the average SiNC diameter, and that from TEM is given by Eq. (1). Data presented as average ± standard deviation.

Raman/nm	In-plane XRD/nm	Out-of-plane XRD/nm	TEM/nm
11.7 ± 0.7	13.1 ± 0.1	30.7 ± 0.1	12.1 ± 4.1

The main source of error in the Raman spectroscopy and XRD results arises from systematic variation of the background signal, which leads to variation in the FWHM of the peak after background subtraction. In TEM, the main source of error is the image resolution error.

**Figure 4:**
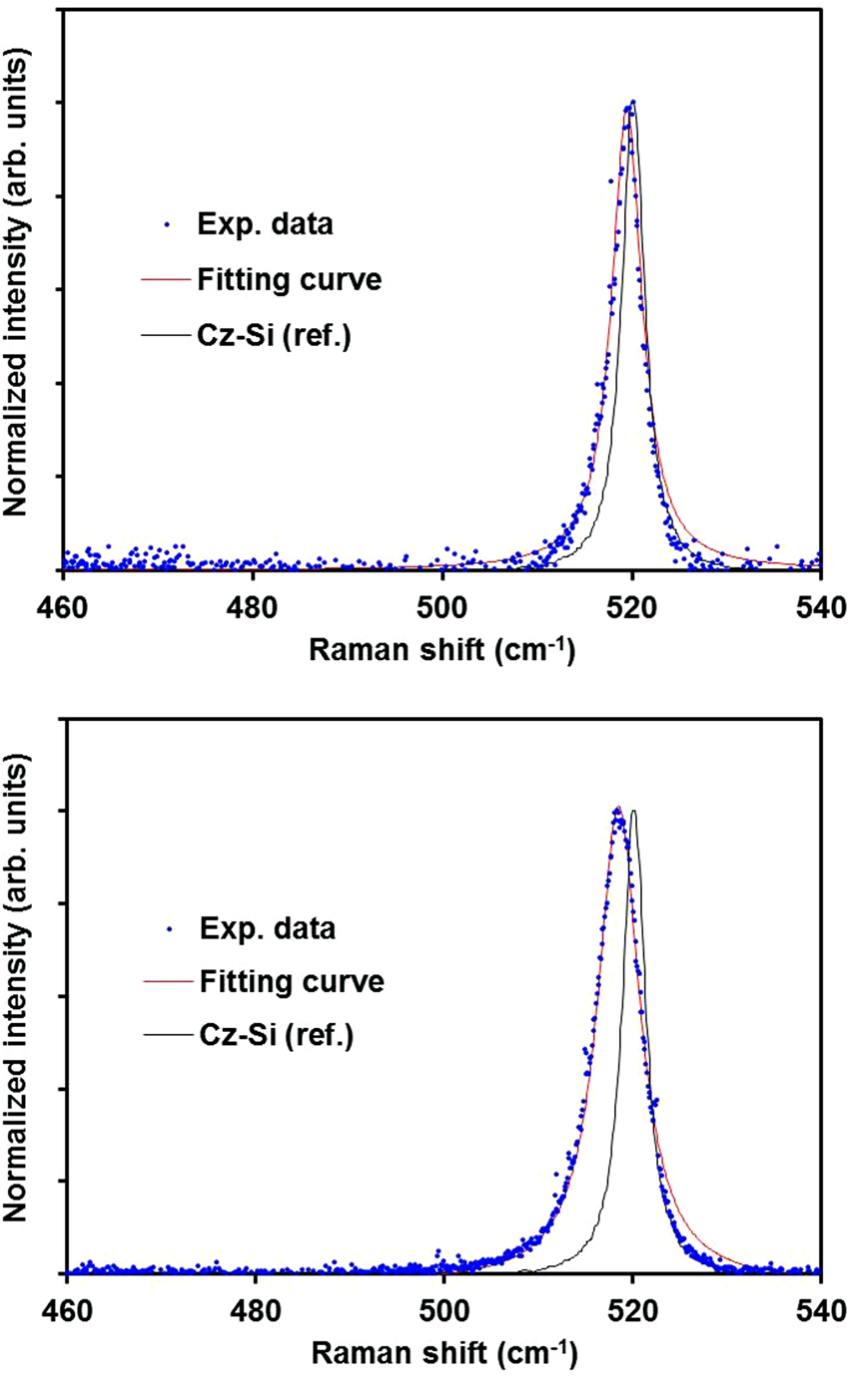
Raman spectra obtained from a-Si thin films thermally annealed at 700 °C for 10 min: (**a**) 30-nm-thick and (**b**) 100-nm-thick films. The spectrum of a Cz-Si wafer is also shown for reference. The peaks are well fit by a Lorentz function, shown in red (coefficient of determination R^2^ > 0.91).

**Figure 5:**
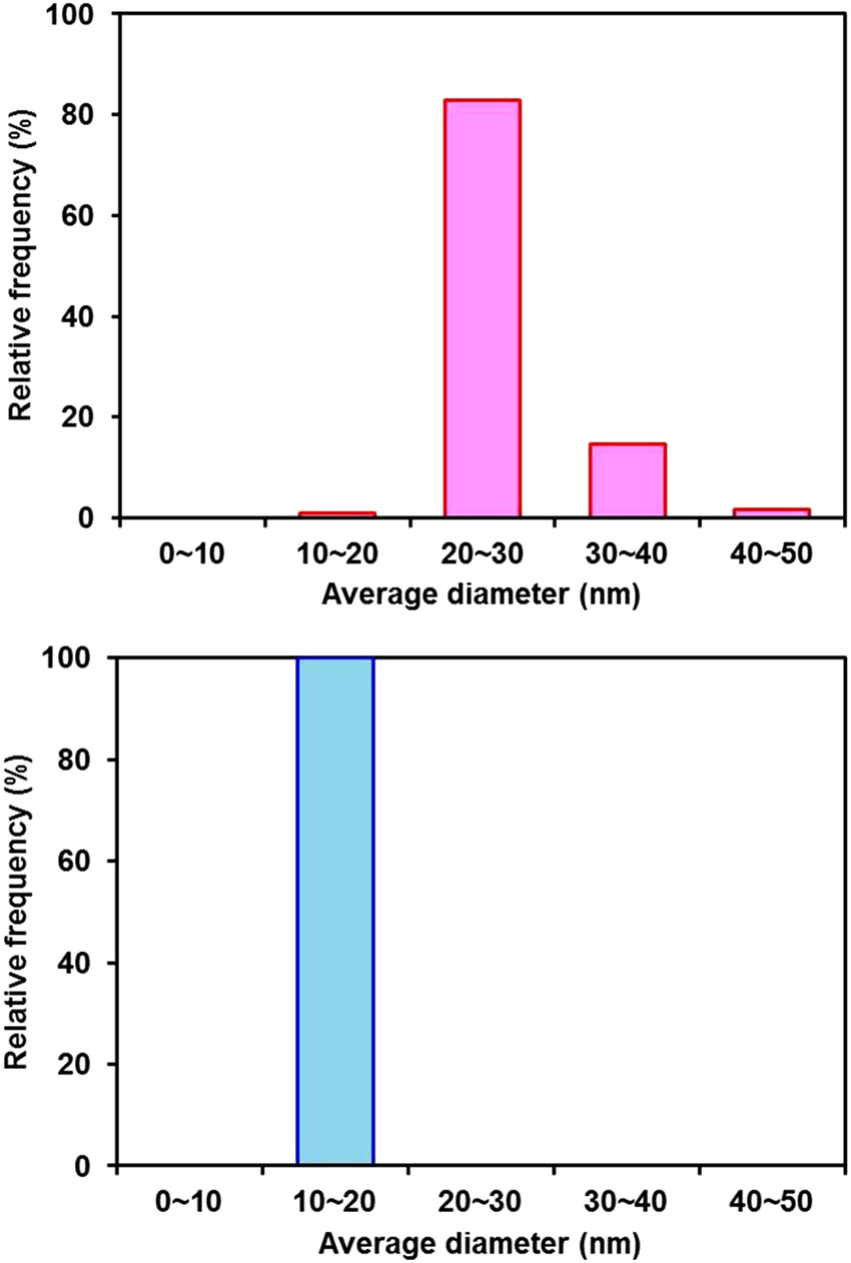
Statistical distributions for the average SiNC diameters. (**a**) 30-nm-thick and (**b**) 100-nm-thick a-Si films thermally annealed at 700 °C for 10 min. The average SiNC diameter was obtained from analysis of the Raman spectra using the KFH model.

**Figure 6:**
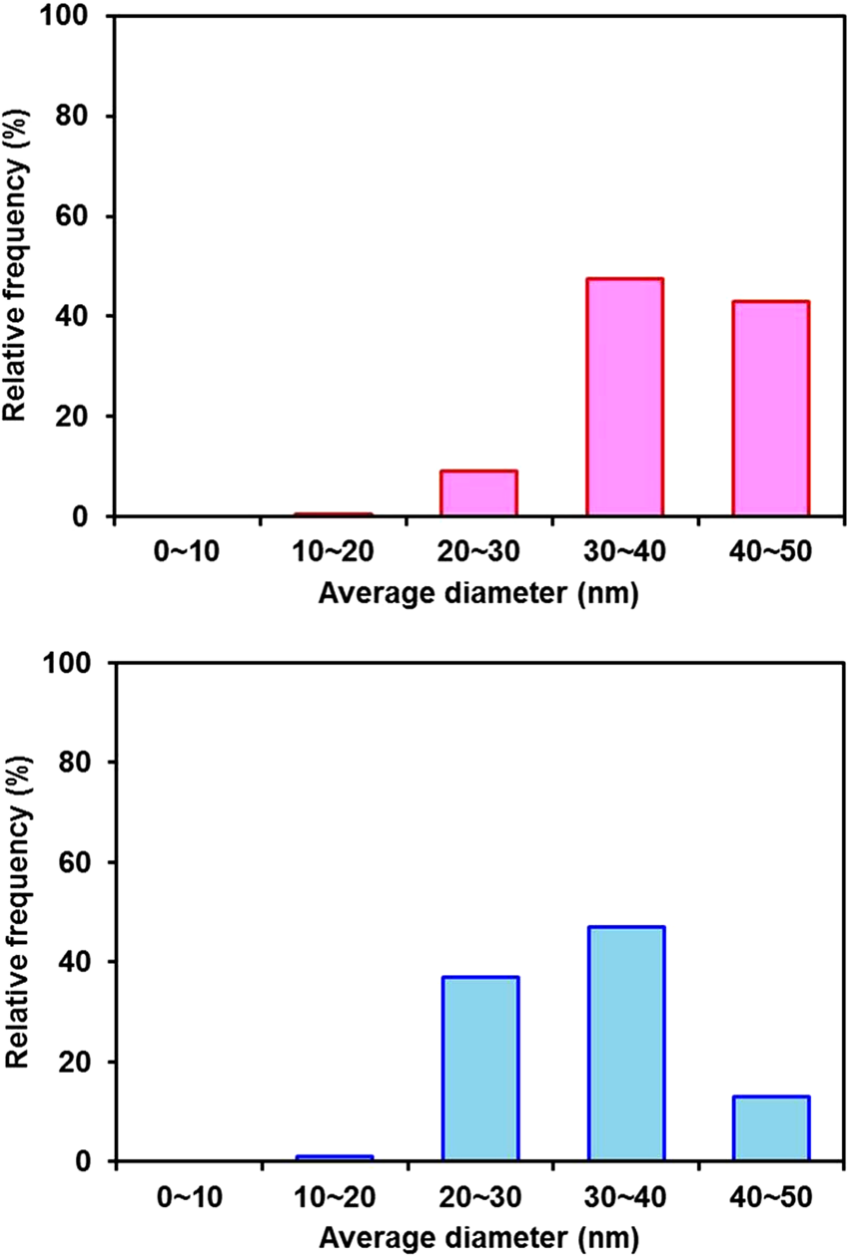
Statistical distributions for the average SiNC diameters obtained from analysis of the Raman spectra using the KFH model. The same a-Si film samples as in Fig. 5 were subjected to annealing at 700 °C for 120 min: (**a**) 30-nm-thick-film and (**b**) 100-nm-thick-film.

**Figure 7:**
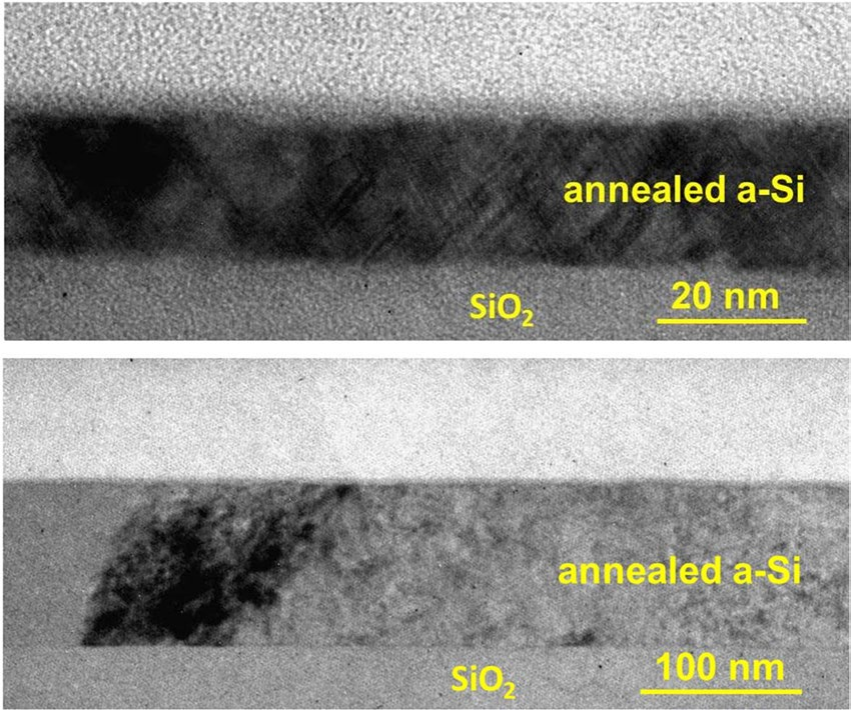
Typical cross-section bright-field TEM micrographs of (**a**) 30-nm-thick and (**b**) 100-nm-thick thermally annealed a-Si films.

The film-thickness-dependent average diameter of the SiNCs after thermal annealing of the a-Si thin films was also investigated. Figure 4 shows a comparison of typical normalized Raman spectra obtained from annealed a-Si thin films and a Czochralski (Cz)-Si reference sample. The main peak near 520 cm^−1^ obtained from the thick film sample is broader than that obtained from the thin film sample and exhibits clear asymmetry. According to the literature^16–18^, a broad asymmetric peak indicates that the thick film sample (100 nm) is composed of small crystallites less than 10 nm in diameter and that the defect density is higher than in a thin film sample (10 nm). The dependence of the defect density on the thickness agrees with the experimental results reported by Zacharias *et al*.^19^, who found that growth faults and dislocations are present in the thicker Si layer. The signal of the TO mode of the amorphous phase at 480 cm^−1^ is not detected, as shown in Fig. 4. This result can be explained as follows. We used the resonant Raman scattering of 364 nm (3.4 eV) light, which corresponds to the first direct electronic transition in Si (Γ_25_ − Γ_15_ = 3.37 eV)^20,21^. In the resonance condition, the signal is greatly enhanced in the crystalline region compared with the amorphous region. Consequently, the resonant Raman spectra are dominated by the signal obtained from the crystalline Si. An interesting feature is that the TO peak for Si crystallite obtained from the thick sample (100 nm) shows a shift to lower wavenumber and asymmetric broadening. Next, the average SiNC diameters were investigated by employing statistical techniques. Figure 5 shows the variation in the average SiNC diameter in terms of relative frequency. It can be seen that the diameters from 20 to 30 nm and 11 to 12 nm are the most frequent in the thin and thick films, respectively. Thus, the average SiNC diameter is higher in the thin film sample than in the thick film sample. To understand the reason for the difference in the average SiNC diameter depending on the film thickness, we analyzed the average SiNC diameters in long-duration annealed a-Si films (700 °C, 2 h). The most important finding is that the most frequent diameters obtained from the thin and thick films were almost the same after long-duration annealing, as revealed by the analysis of the Raman spectra shown in Fig. 6. The dependence of the average SiNC diameter on the thickness in the early stage of annealing can be explained by nucleation and nuclei growth processes^12^. As shown in Fig. 7, the cross-sectional bright-field TEM images suggest that nucleation starts in the a-Si films away from the a-Si/SiO_2_ interfaces. Because the nucleation density per unit volume in the films is independent of film thickness, the amount of nucleation increases with increasing film thickness. Another possible reason for the thickness dependence of the average SiNC diameters is the thermal conductivity of the thin films as a function of film thickness. Films of greater thickness generally have higher thermal conductivity^22^. Because thicker films are heated faster, relaxation cannot be completed, and resulting in crystalline defects or strained bonds. It is therefore thought that these behaviors decrease the average SiNC diameter in thicker films during the early stage of annealing. In contrast, long-duration annealing reduces the density of crystalline defects due to Si-Si bond relaxation. Finally, the most frequent SiNC diameter in thin and thick films increase to almost the same magnitude. Hence, the thickness dependence of the average SiNC diameter is more pronounced in the early stage of crystallization.

In conclusion, we demonstrated a Raman spectroscopy technique that has the capability to characterize the average diameter of SiNCs over different spatial regions and that reveals the spatial heterogeneity in thermally annealed a-Si films. It was found that the a-Si thin film thickness affects the average SiNC diameter in the early stage of the crystallization process. However, the Raman spectra presented in this report suffer from the drawbacks of impulse noise (e.g., noise due to high-energy cosmic rays)^23^ and an interfering background signal originating from the optical elements in the laser light path, which are inevitably brought into the Raman spectrum. The spectral analysis therefore requires removing the impulse noise and subtracting the background signal, which can be prone to error. Nevertheless, the results of this study show that our Raman technique offers not only good accuracy but also a time-efficient method for the determination of average SiNC size.

## Methods

**Figure 8:**
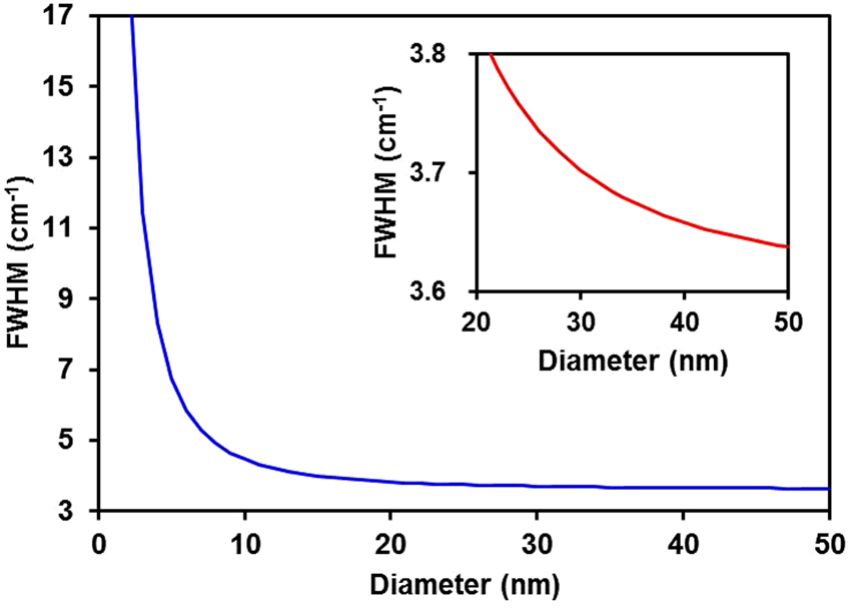
Calculated values for the FWHM as a function of nanocrystal diameter for silicon using the KFH model. The natural linewidth in the Raman spectrum of bulk silicon at room temperature Γ_0_ = 3.6 cm^−1^. Inset: Range from 20 to 50 nm.

Two different a-Si thin films with thicknesses of 30 nm and 100 nm were deposited on 100-nm-thick thermal oxide layers by low-pressure chemical vapor deposition using silane as the precursor gas. After deposition, both samples were thermally annealed at 700 °C for 10 min or 700 °C for 120 min in nitrogen atmosphere for solid-phase crystallization. The room-temperature Raman spectra were measured in a backscattering geometry at a constant spectral resolution of less than 0.1 cm^−1^, as discussed in ref.^8,9^. The samples were excited using the 364 nm line of an Ar ion laser. The superior signal obtained at 364 nm can be attributed to the resonance effect due to the wavelength approximately corresponding to the E1 gap of Si^19^. To obtain statistically significant results, Raman spectra were obtained at 512 points along a 100 μm line at intervals of approximately 200 nm by combination of quasi-line excitation sources obtained from a galvano-mirror and a two-dimensional CCD detector system, as shown in Fig. 1. The power of the laser was 10 mW over 512 points, which was lower than the threshold power for the Fano effect^24^. Thus, artificial spectral broadening and redshifting due to laser irradiation can be neglected. Rayleigh scattering was used as a standard for the *in situ* calibration of the wavenumber. Rayleigh scattering can be detected by separating the concave mirror (dual focusing mirror) in the spectrometer into two parts and was obtained using the same CCD detector simultaneously. The Raman spectra from the CCD data were processed automatically using custom-made software (Princeton Instruments). The background signal was subtracted, the baseline was corrected, and the impulse noise was removed. Impulse noise typically appeared in a maximum of 20 spectra among the 512 spectra, which resulted in poor fitting. Curve fitting of the spectra was performed by means of the least-squares method, assuming that all spectra were composed of Lorentzian-shaped peaks. The KFH model^10^, which is an extension of Faraci’s model^25^, was used to analyze the measured Raman spectra. The natural linewidth Γ_0_ = 3.6 cm^−1^ of bulk Si at room temperature and size distribution parameter σ = 0.3 were used in the analysis. Figure 8 shows the results of the Raman FWHM calculated with the KFH model. The inset shows the range from 20 to 50 nm. The effects of phonon confinement on SiNCs are significant when the SiNC size is smaller than 20 nm, as previously reported^25^. The sensitivity of our Raman technique was on the order of 0.1 nm for SiNC sizes smaller than 20 nm. Although changes in the FWHM in the Raman spectra become less sensitive as the SiNC diameter increases, the Raman technique used in this experiment can detect SiNC diameters of up to 50 nm owing to its high spectral resolution of less than 0.1 cm^−1^. However, for SiNC sizes greater than 30 nm, the uncertainty of the size estimation was ±10 nm. The microstructure of the films was also investigated by XRD and TEM. XRD measurements with both out-of-plane (synchronous scan of 2θ and ω) and in-plane (synchronous scan of 2θχ and Φ) configurations were performed using monochromatic Cu Kα radiation at 45 kV and 200 mA. TEM investigations were performed at 200 kV. As shown in Fig. 3, the Si (111) diffraction peak centered at 2*θ* = 28.4° was observed, which indicates unstrained Si films^26^. Therefore, we can exclude stress-related shifts in the analysis of the Raman spectra. Using the fundamental parameter approach^27^, the SiNC diameter was confirmed.

## References

[1] Priolo F, Gregorkiewicz T, Galli M, Krauss TF (2014). Silicon nanostructures for photonics and photovoltaics. Nature Nanotech..

[2] Heitmann J, Müller F, Zacharias M, Gösele U (2005). Silicon nanocrystals: Size matters. Adv. Mater..

[3] Künle M (2010). Si-rich a-SiC:H thin films: structural and optical transformations during thermal annealing. Thin Solid Films.

[4] Sun W (2015). Switching-on quantum size effects in silicon nanocrystals. Adv. Mater..

[5] Puthen-Veettil B, Patterson R, König D, Conibeer G, Green MA (2014). The impact of disorder on charge transport in three dimensional quantum dot resonant tunneling structures. J. Appl. Phys..

[6] Ferrari AC, Basko DM (2013). Raman spectroscopy as a versatile tool for studying the properties of graphene. Nature Nanotech..

[7] Schlücker S, Schaeberle MD, Huffman SW, Levin IW (2003). Raman microspectroscopy: a comparison of point, line, and wide-field imaging methodologies. Anal. Chem..

[8] Ogura A (2009). Evaluation of local strain in Si using UV-Raman spectroscopy. Mater. Sci. Eng. B.

[9] Ogura A (2008). Evaluation of poly-Si thin film crystallized by solid green laser annealing using UV/visible Raman spectroscopy. J Mater Sci: Mater Electron.

[10] Ke W, Feng X, Huang H (2011). The effect of Si-nanocrystal size distribution on Raman spectrum. J. Appl. Phys..

[11] Neuschitzer M (2012). Grazing-incidence in-plane X-ray diffraction on ultra-thin organic films using standard laboratory equipment. J. Appl. Cryst..

[12] Spinella C, Lombardo S, Priolo F (1998). Crystal grain nucleation in amorphous silicon. J. Appl. Phys..

[13] Thompson CV (2000). Structure Evolution During Processing of Polycrystalline Films. Annu. Rev. Mater. Sci..

[14] Eaglesham DJ, White AE, Feldman LC, Moriya N, Jacobson DC (1993). Equilibrium shape of Si. Phys. Rev. Lett..

[15] Kang D, Kim H, Ali A, Yoon Y, Cho IH (2016). Analysis of the current path for a vertical NAND flash cell with program/erase states. Semicond. Sci. Technol..

[16] Smit C, Van Swaaij RACMM, Donker H, Petit AMHN, Kessels WMM (2003). & Van de Sanden, M. C. M. Determining the material structure of microcrystalline silicon from Raman spectra. J. Appl. Phys..

[17] Campbell IH, Fauchet PM (1986). The effects of microcrystal size and shape on the one phonon Raman spectra of crystalline semiconductors. Solid State Commun..

[18] Richter H, Wang ZP, Ley L (1981). The one phonon Raman spectrum in microcrystalline silicon. Solid State Commun..

[19] Zacharias M (1999). Thermal crystallization of amorphous Si/SiO_2_ superlattices. Appl. Phys. Lett..

[20] Paillard V (1998). Resonant Raman scattering in polycrystalline silicon thin films. Appl. Phys. Lett..

[21] Renucci JB, Tyte RN, Cardona M (1975). Resonant Raman scattering in silicon. Phys. Rev. B.

[22] Wang X, Huang B (2014). Computational Study of In-Plane Phonon Transport in Si Thin Films. Sci. Rep..

[23] Hill W, Rogalla D (1992). Spike-correction of weak signals from charge-coupled devices and its application to Raman spectroscopy. Anal. Chem..

[24] Gupta R, Xiong Q, Adu CK, Kim UJ, Eklund PC (2003). Laser-Induced Fano Resonance Scattering in Silicon Nanowires. Nano Lett..

[25] Faraci G, Gibilisco S, Russo P, Pennisi AR (2006). Modified Raman confinement model for Si nanocrystals. Phys. Rev. B..

[26] Comedi D (2006). X-ray-diffraction study of crystalline Si nanocluster formation in annealed silicon-rich silicon oxides. J. Appl. Phys..

[27] Cheary RW, Coelho A (1992). Fundamental Parameters Approach to X-Ray line-Profile Fitting. J. Appl. Crystallography.

